# Integrated genetic and metabolic characterization of Latin American cassava (*Manihot esculenta*) germplasm

**DOI:** 10.1093/plphys/kiad269

**Published:** 2023-05-06

**Authors:** Laura Perez-Fons, Tatiana Maria Ovalle, Margit Drapal, Maria Alejandra Ospina, Anestis Gkanogiannis, Adriana Bohorquez-Chaux, Luis Augusto Becerra Lopez-Lavalle, Paul David Fraser

**Affiliations:** Department of Biological Sciences, Royal Holloway University of London, TW20 0EX Egham, UK; Alliance of Bioversity International Center and International Center for Tropical Agriculture (CIAT). Km 17, Recta Cali - Palmira, Apartado Aéreo 6713, Cali, Colombia; Department of Biological Sciences, Royal Holloway University of London, TW20 0EX Egham, UK; Alliance of Bioversity International Center and International Center for Tropical Agriculture (CIAT). Km 17, Recta Cali - Palmira, Apartado Aéreo 6713, Cali, Colombia; Alliance of Bioversity International Center and International Center for Tropical Agriculture (CIAT). Km 17, Recta Cali - Palmira, Apartado Aéreo 6713, Cali, Colombia; Alliance of Bioversity International Center and International Center for Tropical Agriculture (CIAT). Km 17, Recta Cali - Palmira, Apartado Aéreo 6713, Cali, Colombia; Alliance of Bioversity International Center and International Center for Tropical Agriculture (CIAT). Km 17, Recta Cali - Palmira, Apartado Aéreo 6713, Cali, Colombia; Department of Biological Sciences, Royal Holloway University of London, TW20 0EX Egham, UK

## Abstract

Cassava (*Manihot esculenta* Crantz) is an important staple crop for food security in Africa and South America. The present study describes an integrated genomic and metabolomic approach to the characterization of Latin American cassava germplasm. Classification based on genotyping correlated with the leaf metabolome and indicated a key finding of adaption to specific eco-geographical environments. In contrast, the root metabolome did not relate to genotypic clustering, suggesting the different spatial regulation of this tissue's metabolome. The data were used to generate pan-metabolomes for specific tissues, and the inclusion of phenotypic data enabled the identification of metabolic sectors underlying traits of interest. For example, tolerance to whiteflies (*Aleurotrachelus socialis*) was not linked directly to cyanide content but to cell wall–related phenylpropanoid or apocarotenoid content. Collectively, these data advance the community resources and provide valuable insight into new candidate parental breeding materials with traits of interest directly related to combating food security.

## Teaser

A characterization of cassava's natural diversity genetic and metabolite levels provides an implemented community resource for future breeding strategies addressing food security and climate challenges.

## Introduction

Cassava (*Manihot esculenta* Crantz) is an important staple food crop for over 800 million people in Africa and South America (Howeler 2013). It has also been recently proposed as a solution to circumvent the global cereal shortages arising (https://www.theguardian.com/global-development/commentisfree/2022/may/12/cassava-nigeria-wean-world-off-wheat). Contrastingly, in Asia, the demand for cassava has changed from a direct food crop to an industrial feedstock being processed into animal feed and starch (Malik et al. 2020). In comparison with other crops, cassava is resilient to environmental fluctuations, it grows on poor soils, and agronomic production does not require sophisticated technology. Thus, it is a good target crop for addressing food and nutritional security concerns in the face of changing climates (Jarvis et al. 2012).

Over the last decade, investments in cassava as a food system have resulted in improved tools and resources for the breeding of new varieties. This has led to the development and deployment of cassava varieties with improved yields (Malik et al. 2020) and nutritional content (Okwuonu et al. 2021), as well as disease and pest resistance (Friedmann et al. 2018). However, despite these notable advances, production levels will not be sufficient to impact the predicted global food and nutritional issues (Ray et al. 2022). In addition, it has become evident that new varieties with improved agronomic traits have experienced inconsistent adoption rates due to altered end-user preferences for various quality traits (Ceballos et al. 2010).

Although modern genomic selection approaches have been incorporated into cassava breeding pipelines, the fundamental strategy still mainly relies on recurrent phenotypic selection, as populations are advanced. Virtually, all cassava cultivation uses clonal propagation and, in most cases, selfing of uncharacterized material. Collectively, this has been shown to cause inbreeding depression. For example, it has been determined that through a single inbreeding generation, over a 60% decrease in fresh root yield can occur (Rojas et al. 2009; Kawuki et al. 2011; Ramu et al. 2017). Similar scenarios have arisen with potato (*Solanum tuberosum* L.) breeding where cultivars are propagated using seed tubers, making the crop recalcitrant to the use of molecular/genomic approaches (Zhang et al. 2021). The potato industry is now adopting a move from clonally propagated tetraploids to true seed-propagated diploids. Sexually propagated species have reduced inbreeding depression, because deleterious mutations are not readily inherited in comparison with clonal propagation (Zhang et al. 2019).

Having well-characterized germplasm collections of cassava that are true to type offers the potential to select suitable parental material for the construction of prebreeding populations capable of delivering diverse quantitative agronomic and quality traits that can be readily selected. Immortalizing these lines also offers the potential for true seed.

Numerous studies have used genomic approaches to genotype existing diversity. Typically, this approach is restricted to local landrace collections or siblings of biparental populations from uncharacterized material (Mbanjo et al. 2021; Ogbonna et al. 2021a). However, collaborative efforts between Asian and Latin American genebank collections have resulted in successful breeding outputs and deployments of stable varieties with consistent phenotypes (Malik et al. 2020). Recently, this strategy has been progressively incorporated into prebreeding programs to deliver biotic resistance in Africa (Perez-Fons et al. 2019; Sheat et al. 2019; Sheat et al. 2021). Among the global cassava germplasm collections available, International Center for Tropical Agriculture (CIAT) offers the largest and most diverse (Howeler 2013; Ferguson et al. 2019). In the present study, genotypic data were used to elucidate the genetic architecture of the population, which was then integrated with agro-ecological profiles, metabolome analysis, and phenotypic data. The metabolome is the biochemical output of the genome and is thus often directly associated with phenotypic traits.

Collectively, the data allow us to better characterize genetic resources, which can be used to select improved parental materials for the construction of prebreeding populations. The outputs have also shed light on the spatial regulation of the cassava metabolome, the influence of agro-ecological adaption on the metabolome, and potential targets for New Plant Breeding techniques to rapidly incorporate traits of interest into suitable cassava metabotypes.

## Results

### Genetic diversity and population structure of LA cassava germplasm collection

**Figure 1.**
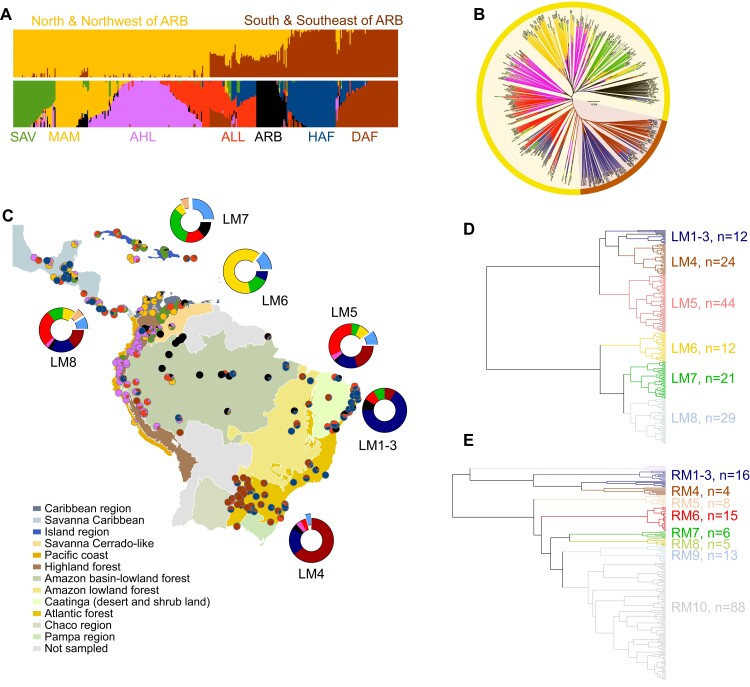
Genetic and metabolic diversity. **A)** Proportion of the genome of each Latin American cassava landrace assigned to each of the 7 subclusters (bottom) grouped under the main genetic gene pools (top). **B)** Group assignment upon the results of the STRUCTURE analysis. Each individual accession is represented by a vertical bar showing the genomic proportion from each subcluster: savanna (SAV), Meso America (MAM), Andean highlands (AHL), Andean lowlands (ALL), Amazon River basin (ARB), humid Atlantic Forest (HAF), and dry Atlantic Forest (DAF). Average proportions of the 7 cassava genetic subpopulations are mapped as filled-in pie-plots over the Central and South America biomes indicated in the figure legend **(C)**. Map generated in ArcGIS. Dendrogram of leaf **(D)** and root **(E)** metabotype classification obtained by hierarchical cluster analysis of LC–MS untargeted data. Clusters of accessions presenting similar metabolite fingerprints are designated as LM or RM clades followed by the number of accessions grouped. Genetic subgroup's composition of LMs is also represented as doughnut plots in **C)**. Additional Asian and African accessions are presented as excised sectors colored as pale blue and pale brown, respectively.

Cassava's genebank held at CIAT station covers a wide diversity of accessions (Supplemental Fig. S1) collected from a range of Latin America locations and biomes. A subset of 481 accessions with complete passport data (Supplemental Data Set 1) was selected for studying the genetic diversity. Bayesian analysis implemented with fastSTRUCTURE was run on the data set without any prior classification to unravel genetic composition, genotype relatedness, and population structure. The clustering method differentiated 2 main gene pools grouping 7 genetic subclusters (Fig. 1A). The genetic subgroups defined as dry and humid Atlantic Forest constituted the cassava's gene pool south and south-east of the Amazon River basin, and the gene pool corresponding to the north and north-western areas of the Amazon River included the genetic subgroups defined as Andean high and lowlands (AHL and ALL), Amazon River basin (ARB), savanna (SAV), and Meso America (MAM). Genetic relatedness based on genetic distance indicates that MAM, SAV, and AHL subgroups are closer to ARB, and the ALL partially clusters with southern subpopulations of the dry and humid Atlantic Forest (DAF and HAF) (Fig. 1B). Noteworthy, a large proportion of the collection remained unclassified due to either unavailable or low quality of sequencing data (Supplemental Data Set 1).

Both gene pools and genetic subgroups co-localize with the different eco-geographic region's characteristic of Central and South America (Fig. 1C). The HAF subgroup includes essentially Brazilian lines with samples from Colombia and Central America (Guatemala, Mexico, and United States), while the composition of the DAF cluster is shared between Brazilian, Paraguayan, Argentinian, and Bolivian landraces. The ARB genetic subgroup comprises accessions from Brazil and Colombia, and the AHL cluster contains varieties from Ecuador, Peru, and Colombia mostly, but also some collected from Bolivia, Mexico, and Caribbean region. The largest proportion of the ALL accessions is from Brazil, Colombia, and Peru, complemented with lines sampled from Ecuador, the Caribbean Islands, Central America, or United States. The SAV subgroup is dominated by Venezuelan, Colombian, and Cuban accessions and minor contributions from Mexico, Panama, Honduras, or Ecuador. Varieties sampled from Colombia, Guatemala, Mexico, Panama, and Costa Rica are the major components of the MAM subcluster, which also contained some lines from Puerto Rico, Jamaica, and Venezuela.

### Metabolic diversity

In the present study, an untargeted metabolomic approach has been used in order to capture the plant's chemical diversity by including all chemical features detected without prior knowledge of their identification.

The biochemical diversity of cassava was evaluated in both uncooked roots and leaves of a germplasm subcollection that included accessions collected from Central and South America and a limited representation of African and Asian varieties. A number of advanced lines developed under CIAT's breeding projects were also part of the diversity panel screened.

Classification of accessions based on chemical fingerprint similarity was generated by hierarchical clustering analysis using untargeted metabolomics data as input matrix (Supplemental Data Sets S2 and S3). The resulting dendrogram differentiated 8 clusters of accessions in the leaf tissue (Fig. 1D) and 10 clusters of accessions in root tissue (Fig. 1E). The groups of accessions were nominated as leaf metabotype (LM) or root metabotype (RM) clades, respectively. The number of cassava accessions was homogenously distributed along the different LM clades, while the number of samples in every RM clade was unequally distributed with RM10 being the largest and concentrating ∼55% of the accessions sampled. In addition, inconsistency between LM and RM classification is evident, and therefore, the implications in relation to genetic diversity and phenotype are subsequently analyzed and discussed separately.

#### Leaf metabolome diversity

The classification of LM clades mirrors the diversity of genetic groups extracted from the single nucleotide polymorphism (SNP) analysis and their corresponding eco-geographic locations (Fig. 1C). Overall, LMs 1 to 5 comprise accessions of the south and south-east Amazon River Basin gene pool, while the bottom branch of the LM dendrogram concentrates those accessions genotyped as north and north-western Amazon River Basin gene pool. LMs 1 to 4 group cassava accessions classified under the HAF and DAF genetic subgroups were mapped in the south and south-east areas of the Amazon River toward the Atlantic coast. In addition, LM5 presents almost equal contributions of ALL accessions and Atlantic Forest accessions collected from these southern regions. Approximately three-quarters of LM6 accessions fall within the MAM genetic subcluster, with Colombian lines from the areas facing the Caribbean Sea being the largest contributors. Landraces collected from Venezuela, Cuba, and Panama form the savanna representatives of LM7, and Brazilian samples contribute to the Amazon sector of this clade. Finally, LM8 shows a similar composition of genetic backgrounds as the southeast cluster LM5, hence, nominated as Mixed-North and Mixed-South clades, respectively. The difference between both mixed LM clades was the geographical locations from where the samples were collected. Leaf clade LM5 is dominated by Brazilian and Colombian landraces, while LM8 includes lines from Venezuela, Guatemala, Peru, and Paraguay. It is noteworthy that African accessions cluster under LMs 7 to 8 and Asian samples spread over LMs 5 to 8 and predominantly in LM7, although genetic classification was not available for these foreign accessions.

The number of chemical features significantly differentiating each LM clade is also annotated in the metabotyping dendrogram (Supplemental Fig. S2). The 2 main South/North branches differ in 403 chemical features, and 50 and 87 mass signals differentiate the subclades within South and North, respectively. Savanna and Meso America accessions (LMs 6 and 7) separate out by 72 differentiating chemical features, and 290 metabolite signals are significantly different between leaf mixed-clades LMs 5 and 8, despite both presenting similar composition of genetic backgrounds.

#### Root metabolome diversity

The metabotyping classification tree based on the analysis of root tissue provided with 10 clades of RMs (Fig. 1E). The RM clades are arranged in 2 main clusters, the top 1 grouping clades 1 to 4 of which over 50% of accessions include humid and dry Atlantic Forest representatives. However, inconsistent alignment of RM clades and genetic subgroups, LMs, or eco-geographical biomes is observed (Supplemental Fig. S3). The exceptions are RMs 6 and 7 of which composition of genetic groups concentrates over 75% of Andean lowlands and savanna accessions, respectively. Nevertheless, the total number of accessions included in RM6 or RM7 represents <10% of the collection. Similarly, RM9 shows similar genetic group composition as RMs 1 to 4 with additional contributors from the ARB subcluster, but no consistency in geographical locations sampled marks the difference between these 2 distant RM clades.

### The construction of a pan-metabolome for cassava

In line with the pan-genome definition, “the entire set of genes from all strains within a clade,” a homologous description is proposed to the pan-metabolome term as the entire set of metabolites present in all natural variants within a clade. Thus, the unbiased nature of untargeted metabolomics together with the wide variety of natural accessions analyzed has enabled the characterization of cassava's pan-metabolome in the present study. Unlike genomic (DNA) information, metabolomic information is tissue specific and, therefore, a spatial metabolomic approach has been followed by analyzing both aerial and storage tissue (leaf and root). The capture of the entire metabolome is feasible, thanks to the operational essence of time-of-flight (TOF) mass analyzers, which facilitates the detection of all chemical components present in the matrix analyzed, as opposed to the studies based on triple quadrupole technologies, which rely on targeted detection of known compounds preselected by the user.

The main bottleneck of untargeted metabolomics data analysis pipelines is the identification and annotation of the chemical entities detected. Here, 2 alternative workflows were followed for metabolite identity characterization of the leaf and root pan-metabolomes. Mining of the leaf metabolome was performed by combining pathway enrichment analysis of significant differentiating features and annotation of untargeted matrix using in-house metabolite libraries validated with chromatographic and mass spectral parameters. This will allow elucidation of diversity at genetic and metabolic level in leaf tissue since both LM clades and genetic groups’ classification mirror each other. On the other hand, root metabolome elucidation was arranged by annotating the topmost abundant molecular features (MF) characteristic of every RM clade using accurate mass measurements.

#### Pan-metabolome of leaf tissue

**Figure 2.**
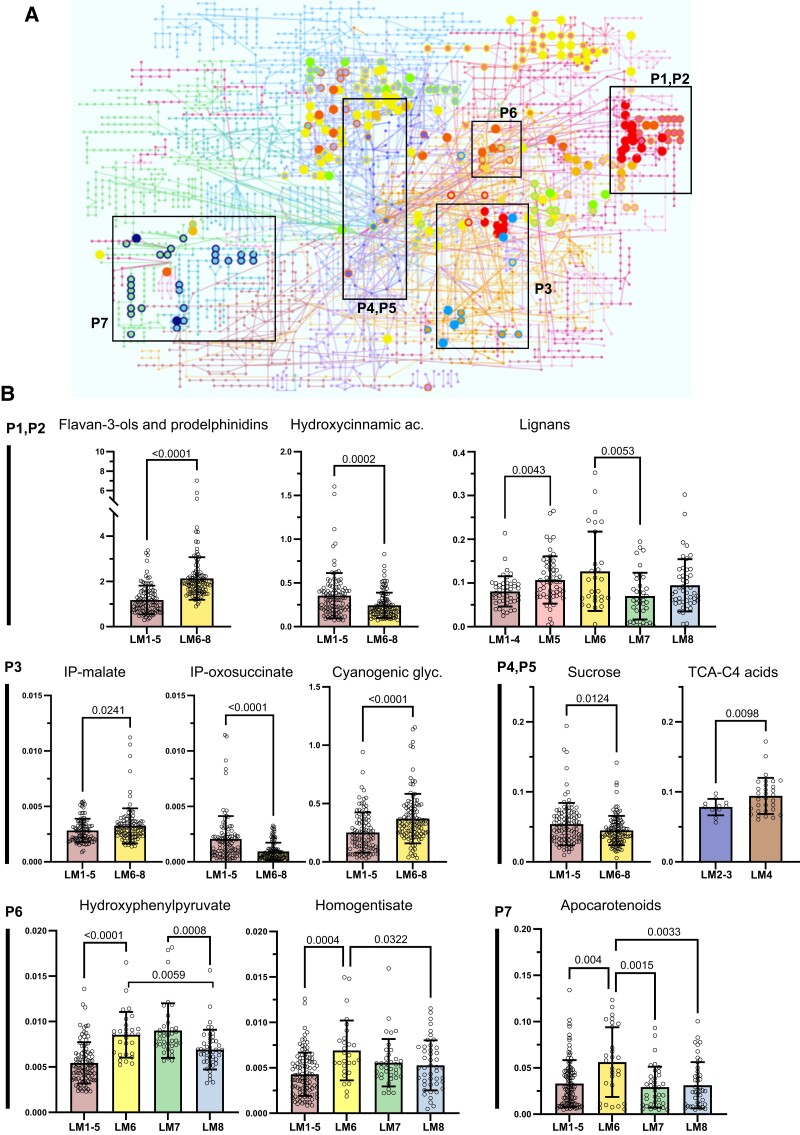
Metabolic sectors differentiating LM clades. **A)** Enriched metabolic pathways differentiating cassava's LM clades mapped onto KEGG's *A. thaliana* metabolic network. **B)** Examples of metabolite's relative amounts of some of the most significantly enriched pathways are displayed as single bar plots representing mean, Sd (error bars), and individual values (open circles). Welch's pair-wise *P*-value is indicated in each graph. P1 and P2, hydroxycinnamic acids, flavonoids, and lignan pathways; P3, valine, leucine, and isoleucine metabolism leading to cyanogenic glycosides biosynthesis; P4 and P5, energy metabolism including sugar metabolism and TCA cycle. C4-acids included malic and fumaric acids. P6, tyrosine metabolism and quinone ring biosynthesis; P7, apocarotenoids. *y* axis represented area metabolite/area internal standard (Ax/Ais). Coloring of clades follows legend of LM dendrogram as displayed in Fig. 1. LM, leaf metabotype clade; ac, acid; glyc, glycosides; and TCA, tricarboxylic acids.

Pathway enrichment analysis was performed using the outputs of pair-wise comparison between the different LM clades individually or in groups as input data (Supplemental Data Set 4). Results of these analysis are summarized in Supplemental Table S1 indicating the most significant enriched pathways, and full detail of all pathways and metabolites enriched per pair-wise comparative is included in Supplemental Data Set 5. Phenylpropanoid and flavonoid super-pathway, cyanogenic glycoside and amino acid biosynthetic precursors, and central metabolism dominate the metabolic differences between North and South Amazon River clusters (Fig. 2A), while apocarotenoid and postchorismate pathway differentiate LM. Accessions from the Southern (LMs 1 to 5) and Northern (LMs 6 to 8) Amazon River regions differ in the composition of hydroxycinnamates, flavan-3-ols, and their polymeric forms (prodelphinidins) (Fig. 2B), but no significant (*P* = 0.61, Welch's *t* test) differences were observed in the flavone and flavonol composition. Condensed tannins prodelphinidins were higher in the Northern LM clades, and an opposite trend was found for their intermediaries, the hydroxycinnamates. Subtle significance was observed in the sucrose levels between the North vs. South LM groups, but stronger significant differences were detected in components of the aliphatic amino acid metabolism valine, leucine, and isoleucine, which ultimately feed into the cyanogenic glycosides biosynthesis of linamarin and lotaustralin (Fig. 2B). Comparisons within LM clades forming South and North dominant groups retrieved distinctive sections of the metabolome too. For example, significant changes in tricarboxylic acid (TCA) cycle–related organic acids were found between representatives of HAF and DAF (LMs 2 to 3 vs. LM4), or different levels of lignans were significant between South-mix clade LM5 and LM1 to 4 accessions (Fig. 2B). Similarly, the lignan content of LM6 was the highest over the rest of LM clades. The clade LM6, largely representing MAM accessions, also presented elevated relative concentrations of apocarotenoids and components of tyrosine metabolism, 4-hydroxyphenylpyruvate (4-HPP), and homogentisate, the latter leading to the biosynthesis of tocopherol's chromanol ring. Finally, salicylic acid and its glycosylated form were significantly more abundant in the Northern LM clades (LMs 6 to 8) (*P* = 0.0004, Welch's test) and the South-mix LM5 compared (*P* = 0.0035, Welch's test) with the rest of Southern clades LMs 1 to 4 (data not shown).

#### Pan-metabolome of root tissue

**Figure 3.**
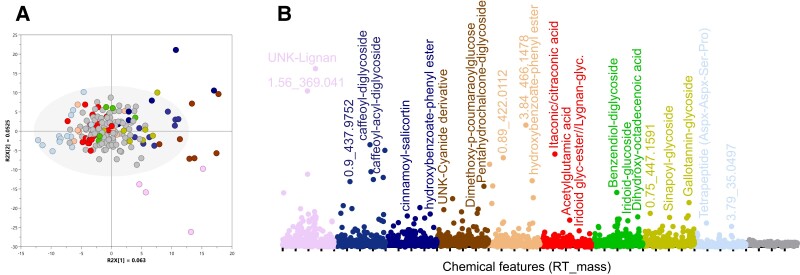
Pan-metabolome of cassava's root tissue. **A)** Score plot of components 1 and 2 of PCA of root's untargeted analysis, including over 500 chemical features detected as variables and 190 accessions as observations. **B)** Scatter plot of chemical features’ relative abundance colored by RM clade. The most abundant features in each clade were annotated after putative identification of mass signals.

The complexity of the chemical composition of root extracts increases from the accessions located at the center of the PCA score plot toward extremes (Fig. 3A). A large group of accessions included in RM10 were characterized by lower chemical diversity, whereas clades RMs 1 to 9 present specific chemotype profiles with both qualitative and quantitative features (Fig. 3B). RM clades 1 to 4 and 5 show the components with the highest accumulation levels. These have been putatively characterized as lignin-type in clade 1 and caffeoyl-glycosides, hydroxycinnamate, and hydroxybenzoate derivatives in RMs 2 to 5 (Supplemental Data Set 6). In addition, RM4 presented higher relative quantities of nitrogenated compounds and glycosyl-chalcone and methoxy-coumaroyl derivatives. In contrast, RMs 6 to 8 contain elevated levels of different monoterpene iridoids and other terpenoid structures in glycosylated form. RM6 also showed higher abundance of certain components involved in the urea cycle, acetyl-glutamic acid, and itaconic/citraconic acids. Additional components found in RM8 were putatively characterized as glycosylated variants of gallotannins and sinapic acid. The metabotype cluster with lowest chemical complexity is represented by RM9 with only 1 tetrapeptide characterized.

### Phenotype–metabolite link per genetic groups

#### Quality traits and metabolome stability

**Figure 4.**
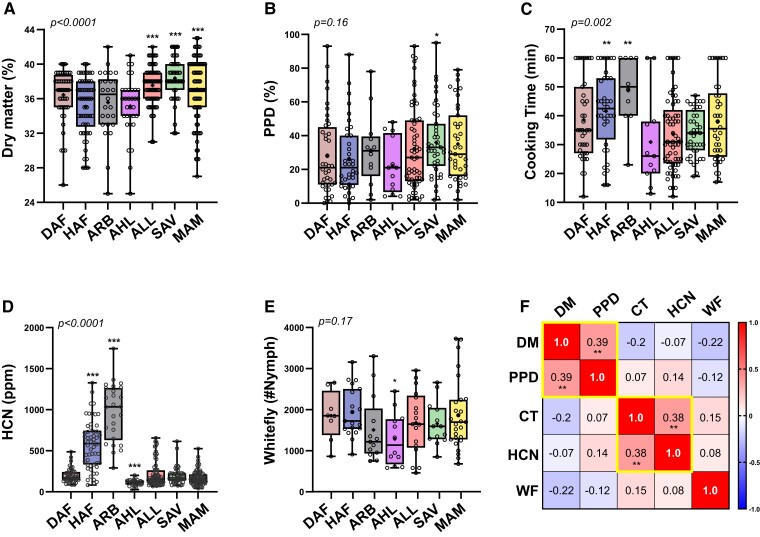
Box plots of phenotyping data collected from 2014 to 2018 at CIAT station. Box extends from the 25th to 75th quartiles and whiskers from the smallest to the largest values. Central line and cross indicate median and mean values, respectively, and accessions' individual values are presented as open circles. **A)** DM, dry matter in % (*n* = 366); **B)** PPD, % of postharvest physiological deterioration of root (*n* = 247); **C)** CT, cooking time of roots in minutes (*n* = 234); **D)** HCN, roots' cyanide content in parts per million (ppm) (*n* = 350); **E)** WF, leaf nymph counts of whitefly A. socialis (*n* = 109); and **F)** matrix Pearson's correlation between phenotypes measured. Yellow boxes denote significant (P < 0.05) correlations. DAF, dry Atlantic Forest; HAF, humid Atlantic Forest; ARB, Amazon River basin; AHL, Andean highlands; ALL, Andean lowlands; SAV, savanna; and MAM, Meso America. Phenotype's ANOVA (one-way) P-value is indicated at the top of every plotting area, and statistical significance of pair-wise comparisons (unpaired t test with Welch correction) between genetic sub groups is also labelled over the corresponding significant group: ***P < 0.001, **P < 0.01, and *P < 0.05.

Phenotypic data concerning dry matter, cooking time, postharvest physiological deterioration (PPD), or root cyanide (HCN) content were recorded between 2014 and 2018, and metabolome stability of tubers was evaluated on material collected from 2016 to 2020 crop years (Supplemental Data Set 1). In addition, a small subset of 110 accessions was tested for whitefly (*Aleurotrachelus socialis*) tolerance as part of CIAT's research program and included in the present study. Genetic subgroups differ significantly in dry matter, cooking time, and cyanide content (Fig. 4, A, C, and D and Supplemental Data Set 7), while no significant differences were detected for PPD or whitefly tolerance (Fig. 4, B and E and Supplemental Data Set 7), the latter likely due to the size of the subset being insufficient for assessing statistical significance. Nonetheless, subtle significant differences were found in the pair-wise comparisons between genetic groups with AHL being the most tolerant to whitefly (lowest count) and the lowest in root HCN. Amazon and humid Atlantic Forest groups presented the highest values of cyanide content and longest cooking times, while Andean lowlands, savanna, and Meso America genetic subgroups show the highest levels of dry matter. Correlation matrix of phenotypic data paired per accessions indicates modest but significant correlation between DM and PPD or HCN and CT (Fig. 4F and Supplemental Data Set 7).

**Figure 5.**
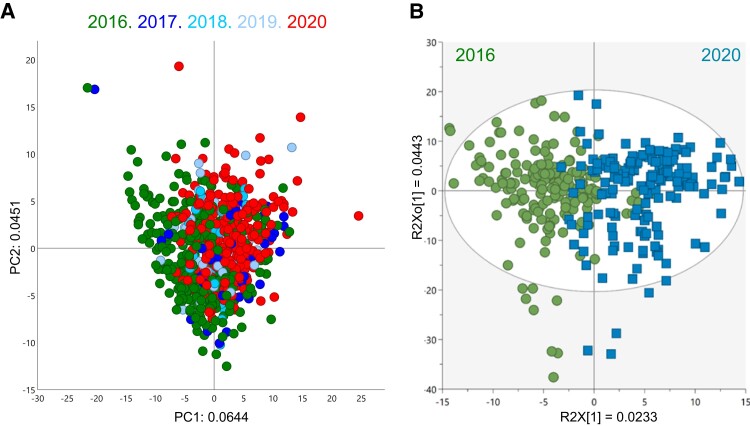
Metabolome stability of cassava roots over multiple crop years. **A)** Score plot of components 1 and 2 of PCA of untargeted metabolite profiling of cassava root accessions harvested from 2016 to 2020. Variation explained by PC1 and 2 indicated in *x* and *y* axes, respectively. **B)** Orthogonal partial least square discriminant analysis (OPLS-DA) score plot comparing accessions harvested in 2016 and 2020. Predictive and orthogonal variations indicated in *x* and *y* axes, respectively.

The PCA score plot including all accessions and crop years explained 10% of variation and shows a consistent overlap between crop years (Fig. 5A). In order to assess metabolite composition stability over the years, a subset of 189 accessions present in both 2016 and 2020 crops was compared using orthogonal partial least square discriminant analysis (OPLS-DA) (Fig. 5B). The OPLS-DA model presented modest fitting (R2) and cross-validation (Q2) values of 0.609 and 0.469, respectively. Variation between classes (years) was 0.0233, and within class (genetic diversity) was 0.0443 (Fig. 5B).

#### Phenotype–metabolite link

Correlations between accessions’ phenotypic records and leaf (Supplemental Data Set 8) and root's chemical features (Supplemental Data Set 9) were attempted as proof-of-concept application of the genetic and metabolome resources generated in the present study.

**Figure 6.**
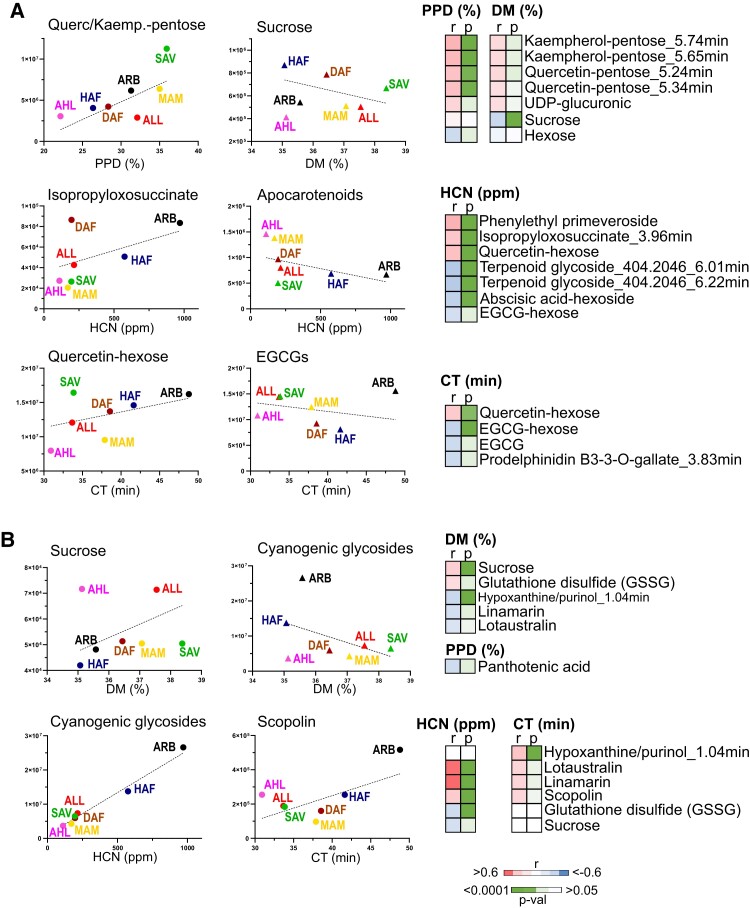
Phenotype–metabolite correlation. Correlation (Pearson) plots between phenotypic values and leaf metabolites **(A)** or root metabolite **(B)** peak areas. Accessions' correlation (*r*) and significance (*P*) values are displayed using heat maps, and graph symbols represent average values of metabolite's peak area per genetic subclusters. Dot symbol was used for positive correlations and triangle for negative correlations graphs. *y* axis represents metabolite's peak area integrated from corresponding mass extracted ion chromatogram. Two terpenoid glycosides with mass 404.2046 detected at different RTs (6.01 and 6.22 min) are included. ppm, parts per million; EGCG, epigallocatechin gallate; Querc, quercetin; Kaemp, kaempherol; DM, dry matter; PPD, postharvest physiological deterioration; HCN, cyanide; CT, cooking time; DAF, dry Atlantic Forest; HAF, humid Atlantic Forest; ARB, Amazon River basin; AHL, Andean highlands; ALL, Andean lowlands; SAV, savanna; and MAM, Meso America.

Pentose derivatives of leaf flavonols quercetin and kaempherol significantly correlate with PPD and DM phenotypes in a positive manner (Fig. 6A), and while negative correlation is observed between leaf-sucrose and hexose, positive correlation is observed between these quality traits and root-sucrose (Fig. 6B). Similarly, levels of linamarin and lotaustralin in roots positively correlate with HCN and CT traits but not with leaf levels of the same cyanogenic glycosides (Figs. 6, A and B). In addition, the flavonol quercetin-hexose and the flavan-3-ol epigallocatechin gallate (EGCG) and its derivatives (hexoside and polymeric forms) in the leaf negatively correlate with both HCN and CT. On the other hand, the coumarin scopolin did not correlate with PPD but with HCN and CT phenotypes in root's tissue, and only pantothenic acid negatively correlated with PPD (Fig. 6B).

Additionally, a number of uncharacterized chemical features in both leaf and root tissue show strong and significant correlation with the different phenotypic traits studied (Supplemental Data Sets S8 and S9).

**Figure 7.**
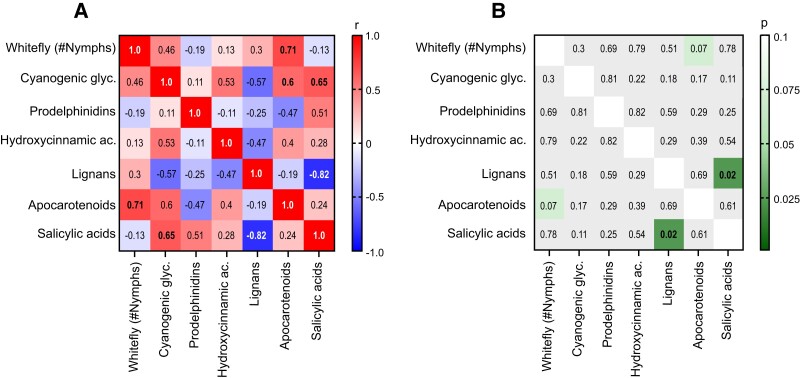
Whitefly phenotype–metabolite correlation. Matrix correlation (Pearson) between whitefly phenotype and metabolites grouped by chemical class **(A)** and corresponding *P*-values **(B)**. Glyc, glycosides; ac, acids; and #, counts.

The correlation approach was utilized to identify potential metabolite markers of whitefly tolerance in the cassava's Latin American collection. No significant correlations were obtained from the analysis of the whitefly phenotype and leaf metabolite chemical classes, except for apocarotenoids. This group of compounds, including ABA and its derivatives and putative blumenols, positively correlate with whitefly nymph count and negatively with prodelphinidins and lignans, although above statistical significance threshold (*P* > 0.05) (Fig. 7). Overall, these results suggest that whitefly tolerance is a complex trait involving multiple metabolome sectors likely modulated by signaling molecules, and therefore, detailed characterization of the biochemical and molecular mechanisms of action would be required to fully elucidate potential phenotype markers.

## Discussion

Numerous genomic studies on cassava collections have been carried out. The populations utilized to date have been limited in their geographical distribution/environments, which has restricted the size of the populations and their diversity analyzed. These criteria have typically resulted in local advanced collections being the focus of analysis. (Ferguson et al. 2019; Drapal et al. 2020; Perez-Fons et al. 2020; Ogbonna et al. 2021a, Ogbonna et al. 2021b; Alves-Pereira et al. 2022; Ocampo et al. 2022) (Supplemental Fig. S1). In addition to genomic approaches, independent metabolomic studies in cassava collections have been focused on selected discovery panels (Drapal et al. 2019; Perez-Fons et al. 2019; Drapal et al. 2020; Perez-Fons et al. 2020). The present study represents one of the largest genomic and metabolomic study performed on cassava to date by exploiting genetic resources present in the CIAT genebank collection, which is the largest and most diverse for this crop worldwide (Reinhardt Howeler et al. 2013; Supplemental Fig. S1). Untargeted liquid chromatography-high resolution tandem mass spectrometry (LC-HRMS/MS) was deployed because of its unbiased nature, mass accuracy, MS/MS capability, and ability to accurately capture wide diversity of chemical features. Thus, in comparison with other analytical apparatus, it provides the closest reflection of the metabolome that can be achieved using 1 analytical platform. The overriding feature of the genotyping data across the CIAT diversity panel was the clustering on agro-ecological location, previously reported in smaller scale studies (Perez-Fons et al. 2020; Ogbonna et al. 2021b; Alves-Pereira et al. 2022; Ocampo et al. 2022). In the case of the leaf metabolome, there was a clear correlation with the genotypic data. This suggests a direct flow of information from gene to metabolite supporting the central dogma of biology. However, the analysis of diversity and stability of the root metabolome indicates that the genetic regulation of this tissue overrides the effect of adaptation pressure and environmental conditions.

The integrative analysis presented in this study also suggests the presence of 2 centers of domestication for cultivated cassava in Latin America, which perhaps evolved from different ancestors and coincided with regions of intensified breeding activity, e.g. Central America, including Caribbean regions, and South-eastern Brazil. While introgression regions from the wild relative Ceara rubber tree (*Manihot glaziovii*) are evident in Brazilian and African germplasms, the original ancestor of Colombian and Northern regions of Amazon basin remain unclear and are still under debate, but presumably from the wild relative *Manihot flabellifolia* (Bredeson et al. 2016; Ogbonna et al. 2021a). Hence, there is the presence of 2 distinctive gene pools within the Latin American collection, each comprising independent metabolome clades of mixed genotypes. It is interesting that cassava diversity evolution follows a similar pattern to the Solanaceae crops such as tomato (*Solanum lycopersicum*) or pepper (*Capsicum* spp.) (Klee and Resende 2020; Tripodi et al. 2021). These proposed domestication centers are coincidental to “The Columbian Exchange” trading points between the New World (Latin America), the Old World (Europe), and the African continent during the 16th century onwards. The data also highlighted the importance of genetic and biochemical adaption to the environments present in these specific agri-geographical locations. Presumably, this phenomenon is one of the reasons why germplasm of Latin America (LA) origin cannot readily be cultivated in other biomes (Alves-Pereira et al. 2022), such as those found in Sub-Saharan Africa. To overcome these limitations but utilize the LA diversity for traits of interest, genetic crossing into an intermediate background could offer potential as precedents exist (Malik et al. 2020). For example, some Latin American accessions classified under the domesticated clade LM8 (e.g. VEN164 and VEN173) have been reported genetically close to the Central East African germplasm or the LA accession CG1320-10 (a cross between MEX1 and PAN51), which is widespread in Africa and genetically clusters under the African breeding germplasm (Ferguson et al. 2019). The genotypes from diverse agro-ecological backgrounds may also offer potential as genetic donors for biotic and abiotic stress resistance predicted to arise from climate change parameters (Alves-Pereira et al. 2022).

Interestingly, classification of the root metabolomes did not reflect the genotypic classification of the leaf metabolomes. This suggests that a different spatial and temporal regulation of the root metabolome in cassava occurs, reflecting its biochemical specialism. In the case of cassava, the tuber is the edible tissue of the plant and thus directly exposed to consumer preferences, which typically relate to quality. One of the issues with present breeding programs utilizing genomic parameters solely is the prevalence of agronomic trait (mostly yield) over consumers’ preference traits. Typically, this is due to poor quality traits and frustratingly arises after time-consuming and expensive breeding cycles. Such findings suggest that a larger metabolomic and/or sensory evaluation would be beneficial to breeding programs. Recently, it has been shown that metabolome selection is an accurate predictor of fruit flavor in tomato complementing genomic selection and sensory traits (Colantonio et al. 2022). Based on these data, the inclusion of metabolomic selection into the breeding program is proposed. In the case of cassava, the tuber metabolome does not correspond with the genotypic classification used in this study but correlation with consumer and agronomic traits has been established (Drapal et al. 2020). Therefore, the inclusion of metabolome analysis and sensory evaluation is necessary if new varieties with acceptable quality traits are to be produced.

The spatial differences in the leaf and root metabolomes across this diverse panel also imply that the population represents a good resource for the study of source–sink genetic elements. The data generated represent an advancement to the genetic/biochemical resources available in cassava. For example, the present study has facilitated the further identification of metabolites by clustering of genotypes and enables future association studies using genome-wide association studies (GWAS) approaches (Zhao et al. 2019; Szymanski et al. 2020; Zhang et al. 2022). A similar approach has been applied in tomato and maize (*Zea mays*) (Hajheidari et al. 2022; Powell et al. 2022). In addition, the study has generated a version of the pan-metabolome for cassava, enabling a core collection of metabolites, which reduces or focuses the number of key MF requiring intense identification and annotation.

The incorporation of selected traits into the data set has enabled the identification of specific clades showing enhanced quality or agronomic traits. The addition of metabolomics data facilitates deciphering the underlying biochemical and molecular mechanism associated with the traits of interest and how this is linked to environmental adaption. One of the examples used is the resistance (or tolerance) of cassava to whitefly infestation. Traditionally, it is accepted that cyanide-containing cassava had evolved as a strategy to alleviate biotic stresses. However, in the case of whitefly infestations, the leaf cyanide content does not correlate with the lowest count of whitefly but with signaling molecules modulating cell wall–related phenolics. The latter corroborates the mechanism postulated previously (Perez-Fons et al. 2019).

In summary, the present data advance the resources in cassava and are an example to other clonal propagated crosses. The accessions characterized can act as parental material in future breeding activities, where the true to type nature can pave the way for the long-term goal of true seed that will reduce future breeding depression by facilitating cultivation from seed stocks. Although there is still much to learn and discover, this study shows the benefits of applying metabolomics to genotypic capture of diversity and potentially subsequent genome-assisted plant breeding. Conversely, it also demonstrates how genomics can have an important impact on expanding our knowledge of biochemical pathways and underlying mechanisms associated with traits. From these large data sets and diversity panel, more selective accessions and subsequent biparental crosses can be generated to decipher underlying mechanisms at the biochemical and molecular levels with higher resolution. Such mechanistic elucidation will aid more rationale design of breeding approaches in the future.

## Materials and methods

### Plant material and growing conditions

Based on the geographical distribution of 3,331 Latin America countries (LAC) landraces held at CIAT cassava world collection, and respective genetic relatedness records, we randomly selected an experimental population of 481 individuals to account for the most heterogeneous and unique cassava (*M. esculenta* Crantz) landraces. These cassava landraces distributed throughout North, Central, and South America were transferred from tissue culture into a screen-house for tissue hardening (Segovia et al. 2012). All agronomic data were collected at CIAT experimental station with an elevation of ∼900 m above sea level. This population was screened for agronomic performance across 4 growing seasons (2014 to 2018). Root and leaf tissue was also collected and immediately frozen in liquid nitrogen for assessing genetic and metabolic diversity as described below. The growing season typically started in May, and harvest occurred the following year in March/April. These trials were designed following a random complete block design with 4 replications (plots); each plot consisted of a total of 16 plants planted in 1 m × 1 m isolation plots and separated by 1 m from each other to ensure consistent plant competition. The agronomic data were collected plant by plant, for the 4 inner most plants, in each plot 11 mo after planting. The plots with <10 geminated plants were removed before data analysis.

### Phenotyping

At harvest, which coincided with 11 mo after planting, 4 innermost plants per genotype were uprooted and used for phenotypic assessments. Roots were separated from the vegetative harvestable biomass (leaves, stems, and original planting stake) and independently weighed. Estimation of dry matter content and cyanogenic potential (HCN) was performed as described by Ospina et al. (2021). For the PPD, we implemented following the methodology described by Luna et al. (2021), and cooking-time experiments were conducted as described by Tran et al. (2021).

### DNA extraction and next-generation sequencing (whole and RAD-Seq)

DNA was extracted according to the cetyl trimethylammonium bromide (CTAB)-based DNA extraction protocol described by Doyle (1991) with minor modifications. The disrupted tissues were incubated at 65°C for 1 h follow by 1 organic extraction using chloroform-isoamyl alcohol (24:1) (*v*/*v*), mixing gently but thoroughly for 30 min at 0°C. The DNA resulting from this extraction was assessed for quality in a 1% (*w*/*v*) agarose gel and quantified using Synergy HT Multi-detection microplate reader (BioTek, USA). RAD-tag libraries were developed by the Beijing Genomic Institute (BGI) following the method described by Baird et al. (2008) using the *Eco*RI restriction enzyme (recognition site: 5′-G/AATTC-3′). The restriction site associated DNA sequencing (RAD-Seq) products from the 355 LAC cassava landraces were processed in the next-generation Illumina sequencing platform HiSeq2000 (BGI, Hong Kong, China).

### Genotyping

The cassava reference genome v6.1 (582.25 Mb arranged on 18 chromosomes plus 2,001 scaffolds) was used (www.phytozome.net) for genotyping. This reference genome included GFF3 files containing functional gene annotations (Prochnik 2012). The GATK pipeline was used to map RAD-Seq reads against the cassava reference genome to discover SNPs and small InDels (DePristo et al. 2011). The pipeline applied Burrows–Wheeler Aligner (BWA) (Li and Durbin 2009) for read alignment with all parameters on the platforms default settings, to resulting outputs being alignments in bam format. The final bam file was sorted according to their genomic locations with SAMtools (Li et al. 2009). For every sample bam file, the HaplotypeCaller tool from GATK produced a genomic variant call format (gvcf), which encoded each sample's variations to the reference genome. Finally, the genotype GVCF tool from GATK aggregated the variations of all sample gvcf files into a single vcf file that encodes the consensus of variants for the sample's cohort. Variants were first filtered in repetitive regions of the reference genome (repeated regions from public catalog present in Phytozome). Next indels, multiallelic, and monomorphic variants were excluded, leaving only biallelic SNP variants in the final data set. Quality filters were then applied to the annotation values calculated by GATK tools. Filter thresholds provided by GATK best practice and exclude variants with values of QD < 2.0, MQ < 40.0, FS > 60.0, MQRankSum < −12.5, and ReadPosRankSum < −8.0. In addition, variants of DP < 3 were filtered out, and this means that each variant should be supported by at least 3 reads. Then, SNPs are filtered for MAF, while the SNP set is formed by retaining variants of at least MAF ≥ 5%. Finally, each set of SNPs is filtered in a manner that kept only variants that produce a data set matrix of at least 90% density (the average missing data for each set being <10%). This was achieved by first sorting the SNPs on decreasing missing data rate and then adding SNPs to the final set (selecting from the top of the sorted list), until the average missing data rate drops below the 10% threshold. These filtering steps produce the final set of 71,540 SNPs. STRUCTURE and PCA were used to analyze genetic structure patterns of the cassava accessions; both analyses were undertaken using 71,540 SNPs. The ΔK method was used to estimate the number of genetic clusters (Evanno et al. 2005). The raw RAD-sequencing reads have been submitted to cassavabase repository (http://www.cassavabase.org/).

### Tissue collection and preparation for metabolite analysis

Leaves and roots were collected separately and immediately frozen in liquid nitrogen. Frozen tissue was then freeze-dried for 2 to 3 d and ground to fine powder as described in Rosado-Souza et al. (2019). Samples were then stored at −20°C until analysis. Due to recurrent frog-skin disease symptoms, affecting leaf quality from 2017 to 2019, only a complete diversity set of leaf material from 2020 was used. The roots from 2016 and 2020 crop years were selected for the present study. Nevertheless, plants presenting healthy roots in 2017 to 2019 crops were included to assess the effect on metabolome stability.

### Metabolite extraction

Ten mg of freeze-dried ground tissue was utilized for extraction of metabolites as described in Perez-Fons et al. (2019). Briefly, 700 *μ*l of 50% (*v*/*v*) methanol was added, and the mixture was shaken for 1 h at room temperature. The addition of 700 *μ*l of chloroform followed by centrifugation (3 min, 14,000 rpm) allowed separation of semi-polar and nonpolar compounds into the epiphase and organic phase, respectively. The semi-polar extract was filtered with 0.45 *μ*m nylon membranes, and the nonpolar extract was dried under vacuum. Both extracts were kept at −20°C until analysis.

### Untargeted metabolomics analysis by liquid chromatography–mass spectrometry (LC–MS)

An aliquot of 95 *μ*l of the semi-polar extract (epiphase) was transferred to glass vials and spiked with 5 *μ*l of internal standard (genistein at 0.2 mg/ml in methanol). Samples were kept at 8°C during analysis, and volume injection was 5 or 1 *μ*l for root and leaf extracts, respectively.

For the analysis of the semi-polar extracts, a C18 reverse phase column and a UHPLC-ESI-Q-TOF system from Agilent Technologies were used. The analytical platform consisted of a 1290 Infinity II liquid chromatograph and a 6560 Ion mobility Q-TOF mass spectrometer operating in Q-TOF mode only and equipped with an Agilent Jet Stream (AJS) electrospray. Data were acquired in MS mode from 100 to 1,700 mDa under negative electrospray ionization. Nebulizer and sheath gas temperatures were 325 and 275°C, respectively; flowrate of drying and sheath gas (nitrogen) was 5 and 12 l/min, respectively, and nebulizer pressure was 35 psi. Capillary VCap, nozzle, and fragmentor voltages were set up at 4000, 500, and 400 V. A reference mass solution was continuously infused to ensure mass accuracy calibration. Compounds were separated in a Zorbax RRHD Eclipse Plus C18 2.1 × 50 mm, 1.8 *μ*m, and 2 different chromatographic methods were optimized for root and leaf tissue, respectively. Roots’ extracts were analyzed with a gradient involving (i) 0.1% (*v*/*v*) formic acid in water and (ii) 0.1% (*v*/*v*) formic acid in 97.5% (*v*/*v*) acetonitrile. Root's chromatographic separation proceeded from 5% B held for 1 min to 30% B in 5 min followed by steep increase to 98% B in 1.5 min. After 1.5 min at 98% B, initial conditions were restored, and column was re-equilibrated for 2 min. Similarly, leaf's chromatographic method used (i) 2.5% (*v*/*v*) acetonitrile in water and (ii) acetonitrile as mobile phase, both solvents containing formic acid (0.03% vol.) as additive. Gradient started at 2% B for 1 min, increased to 30% B over 5 min, stayed isocratic for 1 min followed by an increase to 90% B in 2 min, and stayed isocratic for another 2 min. Initial conditions were restored and re-equilibration lasted 3 min. Flowrate and column temperature of both chromatographic methods were set at 0.3 ml/min and 30°C, respectively.

### Processing of LC–MS data files: extraction of chemical features

Retention time (RT) alignment (maximum time shift ±0.2 min) and extraction of chemical features were performed by using Agilent's Profinder (version 10.0) software in batch recursive mode. The following settings were selected to extract MF within a RT range of 0.3 to 12 min: peak height threshold 1,000 counts, RT tolerance ±0.15 min, mass tolerance 10 ppm, chlorine and formic acid adducts, and water neutral losses were also considered. Only MF with matching scores higher than 70 and present in at least 70% of each sample group [quality control (QC) and samples] were included in the final data matrix. This resulted in the detection of over 500 MF extracted (MFE) in root and over 2,500 MFE in leaf. Putative characterization of chemical identity was inferred from accurate mass values calculated from mass-to-charge ratio (*m*/*z*) signals. Chemical formulae were generated using the following elemental constraints: C, 70; H, 140; O, 40; N, 10; S, 5; and P, 3 (Ma et al. 2014), (https://pmn.plantcyc.org/CASSAVA/search-query?type=COMPOUND&formula=C), formic acid and chlorine adducts, and/or multiply charged species (*z* = 1, 2). Those chemical formulae (up to 5) with the highest score (based on mass difference and isotopic pattern fitting) were selected for blasting against ChemSpider and Dictionary of Natural Products chemical databases. Additionally, an in-house library of cassava metabolites based on chromatographic parameters (RT) and fragmentation pattern (MS/MS) (Perez-Fons et al. 2019) was used to complement and validate findings of the putative identification pipeline described above.

### Data processing and statistical analysis

Batch correction of LC–MS untargeted data (extracted ion chromatogram peak areas) was applied using QC samples. In addition, normalization against area of internal standard was performed. Missing values were input by using the median value of each mass reported from the extraction chemical feature pipeline, and those presenting over 75% of missing values were excluded from analysis. The resulting data matrix was then used as input for multivariate analysis [principal component analysis (PCA), hierarchical clustering analysis (HCA), and orthogonal partial least square discriminat analysis (OPLS-DA)] in SIMCA v17 (Sartorius AG, Germany) and univariate analysis *(t* tests, ANOVA, and Pearson's correlation) in Prism v9.4.0 (GraphPad software LLC). Centering, univariate (in PCA), and pareto-scaling (in OPLS-DA) were applied for multivariate analysis. Pair-wise comparison of LM clades were performed by multiple 2-sample *t* test assuming unpaired data, Gaussian distribution (parametric), and inconsistent Sd (Welch test). Multiple comparisons were corrected with Holm–Sidak post hoc test setting a threshold value for significance (alpha) at 0.01. The adjusted *P*-values obtained from the multiple *t* test analysis and the corresponding *m*/*z* values of chemical features were used as input data for pathway enrichment analysis using the Functional Analysis module (MS peaks to pathways) in MetaboAnalyst v5.0 (Li et al. 2013). Settings selected were negative mode, 10 ppm of mass tolerance, and *P*-value cut-off of 0.05 for the *Mummichog* algorithm. The pathway library selected was Arabidopsis (*Arabidopsis thaliana*) from Kyoto Encyclopedia of Genes and Genomes (KEGG).

Relative amounts of metabolites validating the outputs of enrichment analysis were plotted in Prism and calculated by dividing metabolite's peak area by internal standard's peak area. One-way ANOVA was applied to assess level of significance assuming unpaired Welch and Brown–Forsythe tests, and multiple comparisons of groups were corrected by a family-wise threshold of 0.05. Same assumptions and tests were chosen for the statistical analysis of phenotypic records.

### Accession numbers

The raw RAD sequencing data from this article can be found in the GenBank/EMBL data libraries under accession numbers PRJNA245184.

## Supplementary Material

kiad269_Supplementary_Data

## Data Availability

All relevant data supporting results presented are provided in supplemental files. Metabolomics data sets of raw (areas) and processed data (normalized) and SNP data are accessible in figshare under the following DOI: Perez, Laura; Fraser, Paul; Drapal, Margit; Bohorquez-Chaux, Adriana; Lopez-Lavalle, Luis Augusto Becerra; Ovalle, Tatiana Melissa; et al. (2022): Cassava Diversity data sets. Royal Holloway, University of London. Dataset. https://doi.org/10.17637/rh.21657083.v1.

## Supplemental data

The following materials are available in the online version of this article.

**Supplemental Figure S1**. Number of cassava accessions present in genebank collections worldwide.

**Supplemental Figure S2**. Number of chemical features differentiating LM clades.

**Supplemental Figure S3**. RM clades mapped over Latin American biomes.

**Supplemental Table S1**. Topmost significant enriched pathways differentiating LM clades.

**Supplemental Data Set 1**. Data passport of accessions and phenotypic records.

**Supplemental Data Set 2**. List of chemical features extracted from LC–MS chromatogram of leaf extracts.

**Supplemental Data Set 3**. List of chemical features extracted from LC–MS chromatogram of root extracts.

**Supplemental Data Set 4**. Multiple *t* test pair-wise comparisons of LM clades.

**Supplemental Data Set 5**. Detailed output of pathway enrichment analysis.

**Supplemental Data Set 6**. Putative identification of the most abundant root chemical features.

**Supplemental Data Set 7**. Results (P-values and r) of statistical analysis performed by comparing genetic groups per phenotype and phenotypes’ correlations as presented in Fig. 4.

**Supplemental Data Set 8**. Leaf chemical feature–phenotypic record correlations.

**Supplemental Data Set 9**. Root chemical feature and phenotypic record correlations.
