# Supplementary material for: Integrated genetic and metabolic characterization of Latin American cassava (Manihot esculenta) germplasm
Source: Plant Physiol. 2023 May 6;192(4):2672–86. doi: 10.1093/plphys/kiad269 (PMC10400033; doi:10.1093/plphys/kiad269)
Supplement: kiad269_Supplementary_Data [file kiad269_supplementary_data.zip › Supplemental Data_Legends.pdf]

Supplemental Data

Integrated genetic and metabolic characterisation of Latin American cassava (*Manihot esculenta* Crantz) germplasm; deciphering natural diversity and its implications for future breeding strategies

Perez-Fons L, Ovalle TM, Drapal M, Ospina MA, Gkanogiannis A, Bohorquez-Chaux A, Becerra Lopez-Lavalle LA, Fraser PD

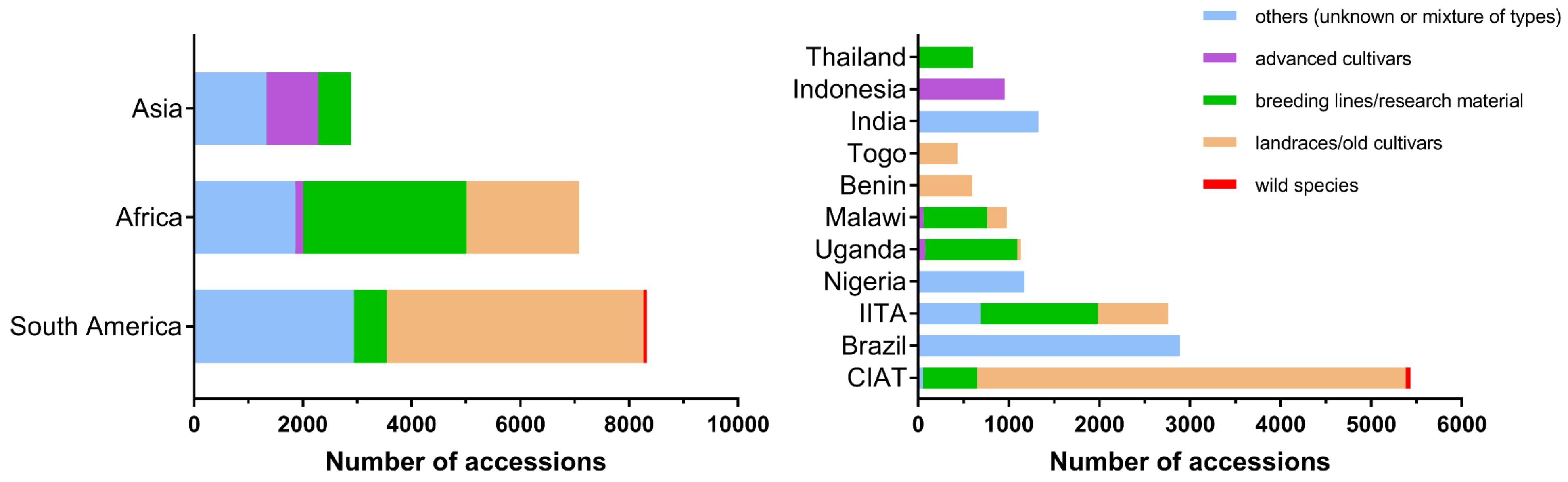

Supplementary Figure S1: Number of cassava accessions present in genebank collections worldwide up to 2010. *Source: FAO. Save and Grow: Cassava (2014); FAO 2010. The second report on the state of the world's plant genetic resources for food and agriculture. Rome.*

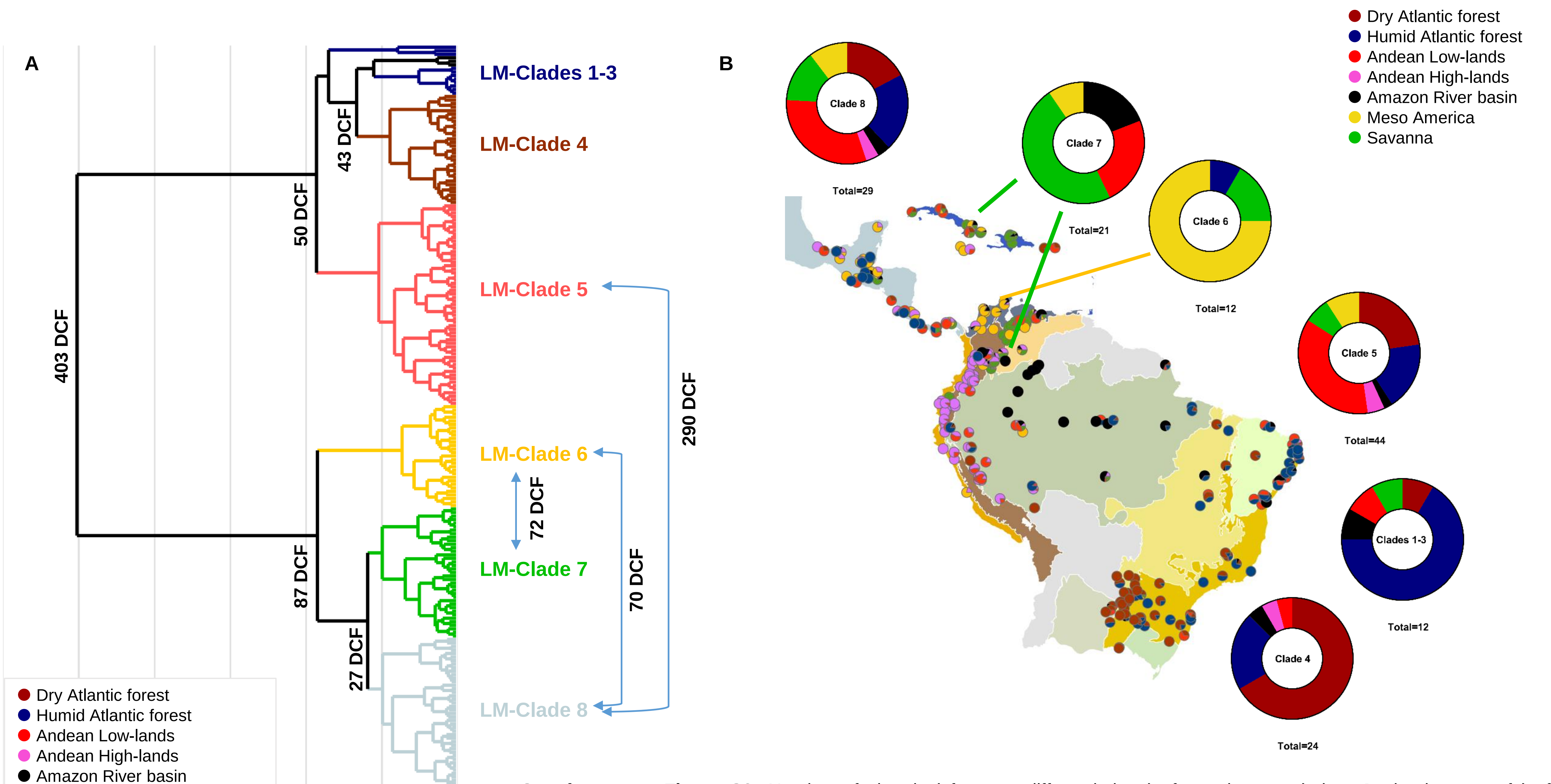

Supplementary Figure S2: Number of chemical features differentiating leaf metabotype clades. **A**, dendrogram of leaf metabotype clades including the number of significant chemical features differentiating each branch. Pair-wise comparison of LM-clades was performed by using multiple t-test analysis and Holm-Sidak post-hoc test (alpha=0.01). **B**, genetic composition of accessions clustered in every leaf metabotype (LM) clade mapped over the cassava genetic sub groups distribution over Latin American eco-geographic regions. DCF: differentiating chemical features.

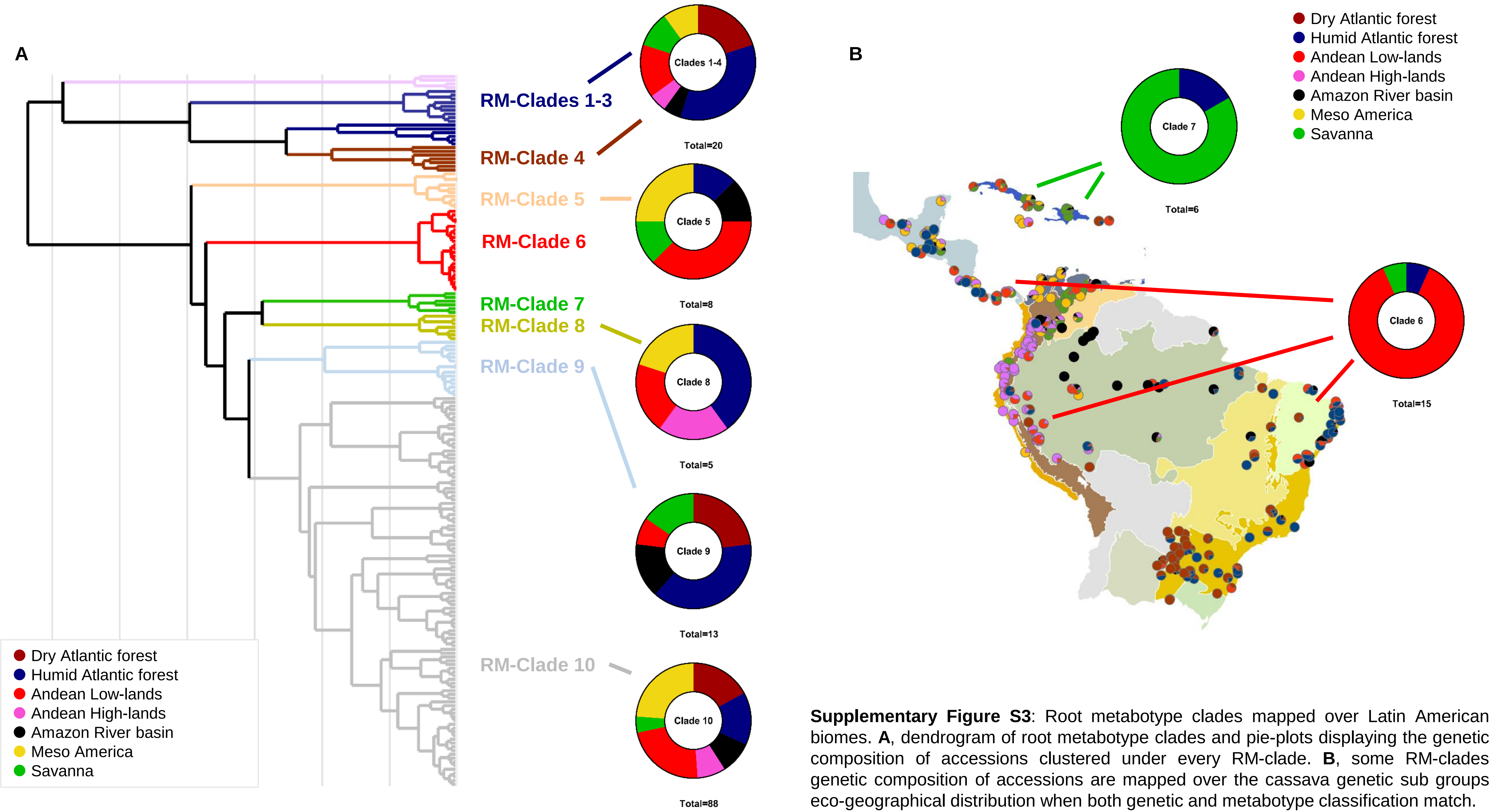

Supplementary Figure S3: Root metabotype clades mapped over Latin American biomes. **A**, dendrogram of root metabotype clades and pie-plots displaying the genetic composition of accessions clustered under every RM-clade. **B**, some RM-clades genetic composition of accessions are mapped over the cassava genetic sub groups eco-geographical distribution when both genetic and metabotype classification match.

Supplementary Table S1: Summary of the most significant enriched pathways differentiating LM clades. Pathway enrichment analysis was performed by using the Functional Analysis module (MS peaks to pathways) in MetaboAnalyst v5.0. #SHE: number of significant hits enriched; #DCF: number of significant differential chemical features obtained by multiple unpaired t-test comparisons with Welch correction and Holm-Sidak post-hoc correction for multiple comparisons, setting an alpha threshold for significance at 0.01.

Pair-wise LM clades comparisons	# DCF	# SHE	Highest significant pathways enriched (ranked by permutation gamma p-value)
1. South (LM1-5) *Vs.* North (LM6-8)	403	241	• Flavonoid, flavone & flavonol, phenylpropanoids & anthocyanin biosynthesis<br>• Val, Leu, Ile biosynthesis & degradation<br>• Sugar metabolism
2. Atlantic Forest (LM1-4) *Vs.* Mix-South (LM5)	50	101	• Riboflavin biosynthesis<br>• Synthesis and degradation of ketone bodies<br>• Lignans biosynthesis
3. Dry (LM4) *Vs* Humid (LM1-3) Atlantic Forest	43	54	• Val, Leu, Ile biosynthesis<br>• Organic acids
4. Meso America (LM6) *Vs*. Savanna & Mix-North (LM7-8)	87	53	• Phenylalanine biosynthesis<br>• Chlorophyll ring biosynthesis<br>• Flavonoids, phenylpropanoids & anthocyanin biosynthesis
5. Meso America (LM6) *Vs*. Savanna (LM7)	72	64	• Quinones biosynthesis<br>• Lignan biosynthesis<br>• Phe, Trp, Tyr biosynthesis
6. Meso America (LM6) *Vs*. Mix-North (LM8)	70	32	• Chlorophyll ring biosynthesis<br>• Flavonoids and phenylpropanoid biosynthesis
7. Mix-South (LM5) *Vs*. Mix-North (LM8)	290	129	• Flavonoids and phenylpropanoid biosynthesis<br>• Chlorophyll ring biosynthesis
8. Savanna (LM7) *Vs*. Mix-North (LM8)	27	33	• Chlorophyll ring biosynthesis<br>• Anthocyanin biosynthesis
